# COPD: maximization of bronchodilation

**DOI:** 10.1186/2049-6958-9-50

**Published:** 2014-10-15

**Authors:** Stefano Nardini, Gianna Camiciottoli, Salvatore Locicero, Rosario Maselli, Franco Pasqua, Giovanni Passalacqua, Riccardo Pela, Alberto Pesci, Alfredo Sebastiani, Alessandro Vatrella

**Affiliations:** Pulmonary and TB Unit, Vittorio Veneto General Hospital, Vittorio Veneto, TV Italy; Department of Experimental and Clinical Medicine, Section of Respiratory Medicine, AOU Careggi, Florence, Italy; Pneumotisiology, NiguardaCa’Granda Hospital, Milan, Italy; Department of Medical and Surgical Sciences, Section of Respiratory Diseases, University “Magna Graecia” of Catanzaro, Catanzaro, Italy; Pneumology Rehabilitation, Villa delle Querce Hospital, Nemi, Rome, Italy; Department of Internal Medicine and Medical Specialities, Respiratory Diseases and Allergology, Università degli Studi di Genova, Genoa, Italy; Pneumology Unit, C. e G. Mazzoni Hospital, Ascoli Piceno, Italy; Department of Pneumology, San Gerardo Hospital Monza (Mi), Monza, Italy; Pneumology Unit, AO S.Camillo-Forlanini, Rome, Italy; Department of Medicine and Surgery, University of Salerno, Salerno, Italy

**Keywords:** Bronchodilation, COPD, Dyspnea, Exercise tolerance, Fixed combination indacaterol/glycopyrronium, HRQoL, Hyperinflation, LABA, LAMA

## Abstract

The most recent guidelines define COPD in a multidimensional way, nevertheless the diagnosis is still linked to the limitation of airflow, usually measured by the reduction in the FEV_1_/FVC ratio below 70%. However, the severity of obstruction is not directly correlated to symptoms or to invalidity determined by COPD. Thus, besides respiratory function, COPD should be evaluated based on symptoms, frequency and severity of exacerbations, patient’s functional status and health related quality of life (HRQoL). Therapy is mainly aimed at increasing exercise tolerance and reducing dyspnea, with improvement of daily activities and HRQoL. This can be accomplished by a drug-induced reduction of pulmonary hyperinflation and exacerbations frequency and severity. All guidelines recommend bronchodilators as baseline therapy for all stages of COPD, and long-acting inhaled bronchodilators, both beta-2 agonist (LABA) and antimuscarinic (LAMA) drugs, are the most effective in regular treatment in the clinically stable phase. The effectiveness of bronchodilators should be evaluated in terms of functional (relief of bronchial obstruction and pulmonary hyperinflation), symptomatic (exercise tolerance and HRQoL), and clinical improvement (reduction in number or severity of exacerbations), while the absence of a spirometric response is not a reason for interrupting treatment, if there is subjective improvement in symptoms. Because LABA and LAMA act via different mechanisms of action, when administered in combination they can exert additional effects, thus optimizing (i.e. maximizing) sustained bronchodilation in COPD patients with severe airflow limitation, who cannot benefit (or can get only partial benefit) by therapy with a single bronchodilator. Recently, a fixed combination of ultra LABA/LAMA (indacaterol/glycopyrronium) has shown that it is possible to get a stable and persistent bronchodilation, which can help in avoiding undesirable fluctuations of bronchial calibre.

## Review

Although the AGENAS guidelines (http://www.agenas.it/images/agenas/pnlg/BPCO.pdf), the inter-societal document (http://www.aimarnet.it/wordpress/up-contente/uploads/2013/11/Gestione-BPCO_04_layout-1-blk.pdf) and the latest GOLD guidelines define COPD in a multidimensional way, for its diagnosis it is still necessary to detect a functional characteristic: the limitation of airflow, usually measured by the reduction in the FEV_1_/FVC ratio below 70%.

Obstructive abnormalities of the small airways with a reduction in their caliber and destructive phenomena of the parenchyma with reduced lung elastic recoil, represent the two pathophysiological mechanisms responsible for airflow limitation.

The fact that the small airways are the compartment where the histopathological damage occurs for the two above cited mechanisms, was demonstrated many years ago in a group of COPD patients who died because of cardiac failure. The prevalence of one of the two histopathological alterations (bronchial inflammation or parenchymal destruction) corresponded to different clinical phenotypes: these were functionally distinguished by a different alteration of the parameters indicative of hyperinflation and impaired gas exchange more than by the parameters indicative of obstruction [1].

Another important pathophysiological consequence of the bronchial tree involvement in COPD is the marked increase in resistance, up to 40 times more than normal, due to the presence of mucus hypersecretion, with obstruction and obliteration of the small airways. The consequence is that the time needed for these obstructed lung units to empty (or wash-out) their volume and to achieve their passive equilibrium point at the end of a normal expiration maneuver, is significantly increased. Many of those units do not reach their relaxation volume before a new inspiration is initiated. As a result, part of the gas that would have been expired in a normal lung, remains “trapped” in patients with COPD causing hyperinflation. This condition is more severe during exercise, when more and more units are unable to empty (or wash-out) their gas, as expiratory time decreases when the respiratory rate increases and such hyperinflation represents the pathophysyological basis of dyspnea on effort that is the most invalidating symptom in COPD.

The critical points of the physiopathological approach to the illness can be summarized in this way:

- the FEV_1_/FVC is an index of bronchial obstruction that does not reflect alterations in the small caliber airways described by pathological anatomy and already present in the first phases of the illness; FEV_1_ does not define the prevalent phenotype of the illness
- the study of the peripheral airways is not easily done in a normal clinical routine
- the distal airway involvement is associated with increased static lung volumes (hyperinflation), as well as destruction of the vascular capillary pulmonary zone and alterations in gas exchange. All these physiopathological alterations together are responsible for the symptoms and clinical course of the illness
- COPD is a complex illness that goes beyond the simple functional definition. The contribution of pathophysiology to this definition cannot, however, disregard to perform a global spirometry test and a diffusion test

### Is bronchial obstruction a necessary and sufficient condition for a COPD diagnosis?

The spirometric evidence of a not completely reversible obstruction is a necessary condition for the diagnosis of COPD which could be better supported by a nitrogen wash-out test. However, the seriousness of the obstruction is not directly correlated to the symptoms or invalidity determined by the COPD; indeed, “similar levels of obstruction can correspond to very different levels of invalidity and prognosis*”* [2].

The seriousness of the patient’s condition appears to be determined not only by the deterioration of pulmonary function, but also by the symptoms, the propensity for exacerbations, the nutritional status, and the presence of other diseases (comorbidities) [3].

Therefore, even COPD’s progression should be evaluated and monitored not only with regards to respiratory function, but also looking at other parameters, such as the type and intensity of symptoms, the frequency and severity of exacerbations, the functional status of the patient, the use of drugs and the quality of life.

Which pathophysiological parameters is bronchial obstruction evaluated on?

1. There are two criteria to evaluate bronchial obstruction: “Fixed ratio” (FEV_1_/FVC <70%) and “Lower limit of normal (LLN, measured value below the 5^th^ percentile of a healthy population, made up of non-smokers).” The first one is more practical, but it is said to generate under-diagnosis in young people and over-diagnosis in the elderly. The second one, could be more precise, but there are few or no reliable estimates of the distribution of the FEV_1_/FVC ratio in the various age ranges [4]. Given that COPD diagnosis - particularly in the elderly - is never only spirometry-based and diagnostic suspicion - generated by symptoms and individual risk-profile - precedes the spirometry, the potential diagnostic error of employing the fixed ratio will be reduced by the clinical evaluation leading to the spirometry test [4].

### Once the patient has been profiled, what is expected from medical therapies?

It is necessary to remember that the three stakeholders of the diagnostic process are: the patient (the central element), the medical team and the health institutions or rather the “taxpayers” Expectations often coincide, but not always are the same. For example, a functional improvement is an objective which is justifiably pursued by the physician, but it does not represent the primary objective for the patient, who is more interested in an improvement in symptoms and in keeping the therapy as simple and safe as possible.

The improvements expected by the patient are therefore an increased tolerance for physical exercise and a reduction of dyspnea, because these reflect positively on daily activities and quality of life. Physical activity and muscular force, already lessened in the initial phases of COPD, continue to decrease as the disease progresses [5–7].

**Figure 1:**
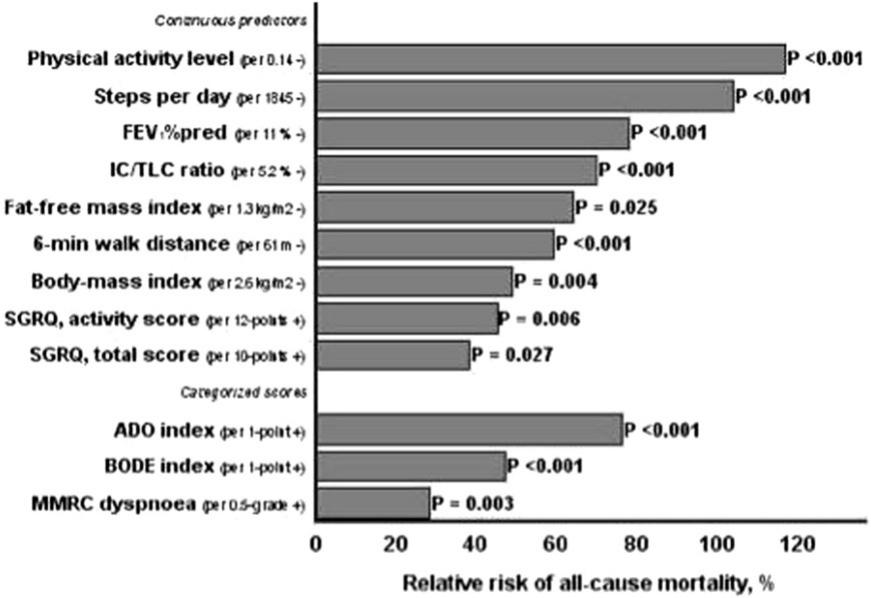
**Comparison between relative risk of death associated with physical activity and established predictors of mortality.**

On the other hand, the reduction in physical activity is the strongest predictor of mortality for all causes in COPD patients (Figure 1) [8] and is correlated among others to dynamic hyperinflation [9].

As a consequence, the reduction of pulmonary hyperinflation increases the tolerance to exercise and therefore physical activity [10, 11]; in this context one could fancy a positive effect on survival, even if at this point there is no scientific evidences to confirm this theory. The improvement in capacity is one of the key results obtained through pulmonary rehabilitation, as stated in abundant literature and recent guidelines (ATS/ERS, Nice) [12, 13], but the use of “desufflating medications”, or rather bronchodilators, amplifies the effects of rehabilitation itself [14].

### How to obtain bronchodilation

#### Routes to obtain bronchodilation

**Figure 2:**
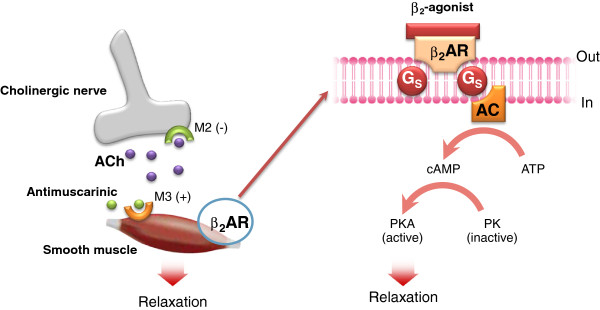
**Mechanisms of bronchodilatory action of antimuscarinic agents and beta2-adrenergic receptor agonists.** Antimuscarinics block the binding of acetilcholine (ACh) to M3 muscarinic receptor, thereby inhibiting smooth muscle cell contraction. Beta2-adrenergic receptor agonists bind to beta2-adrenergic receptor and induce a cascade of signal transduction events that ultimately lead to smooth muscle relaxation.

The reduction of resistance in the airways depends on factors both intrinsic and extrinsic to them, being constituted by smooth muscle relaxation, reduction of inflammation of the bronchial and bronchiolar wall, and reduction of secretions.Airway smooth muscle relaxation can be obtained by two main pharmacologic strategies: directly through stimulation of β2-adrenoceptors (β2-ARs) with β-agonists or indirectly by inhibiting acetylcholine signaling via muscarinic receptors with muscarinic antagonists (Figure 2).

The first one is based on the use of β2-adrenoceptors agonists, that, by binding to the β2-adrenoceptor receptors on the surface of the smooth muscle cells in the airways, including small ones, directly determine the relaxation and the consequent bronchodilation [15].

The cellular mechanism at the basis of bronchodilation involves the activation of the adenylcyclase and the generation of intracellular cAMP, which then activates the effector molecules protein-kinase A (PKA) and Epac (effector of cAMP).

In turn, the PKA works through phosphorylation of the proteins that control smooth muscle tone, the Epac induces the relaxation of the smooth muscle independently of PKA and the cyclical AMP sequesters the intracellular Ca, all of this having, as a consequence, smooth muscle relaxation [16, 17].

**Figure 3:**
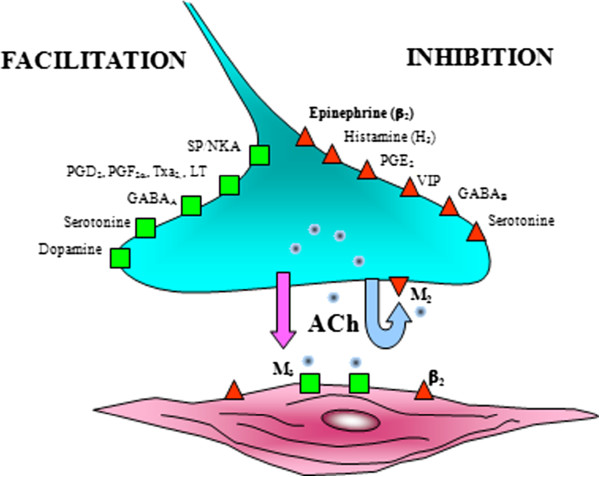
**Presynaptic mediator involved in ACh release (neurojunctional plaque).** Modified from [71, 72].

The second one is based on the use of antimuscarinics, which act indirectly, competitively antagonizing the receptors of the contractile agonist (ACh).The parasympathetic activity in the airways is, in fact, mediated by the muscarinic receptors M1 and M3, whose stimulation produces the contraction of the smooth muscle, mucus secretion and increase in ciliary activity, and from the M2 receptors, which instead inhibit the release of ACh from the nerve endings [Figure 3].

The increase in cholinergic bronchomotor tone is considered important to the pathogenesis of COPD, contributing to the increase in bronchial obstruction through bronchoconstriction and mucus hypersecretion [18, 19].

β2-adrenergic receptor agonists bind to β2-ARs on the surface of smooth muscle cells at all airway levels, even in the small airways involved in COPD. Antimuscarinic agents antagonize muscarinic (M3) receptors on airway smooth muscle and thereby prevent smooth muscle contraction. Though a direct vagal innervation is absent in the small airways, muscarinic receptors are present in the small airways and are likely activated by extra neuronal acetylcholine in diseases such as COPD. Therefore, antimuscarinic agents could dilate both large and small airways. The traditional separation of β2-agonists acting on the distal airways and antimuscarinics acting on the proximal airways is likely to be revisited and a combination therapy with both classes of drugs may provide greater bronchodilation at all airway levels than either component alone. Two different mechanisms of bronchoconstriction have the potential to maximize the bronchodilator response and can help to overcome the inter- and intra-patient variability in response to individual agents often observed in patients with COPD. Furthermore, it can be expected that an antimuscarinic agent together with a long-acting β-2, “thus combining different mechanisms”*,* can have a synergistic effect on tolerance to physical activity, on daily activities and on the quality of life which, as stated before, is also linked to simple administration of drugs.

Analyzing the literature regarding the most recent bronchodilators available for the treatment of COPD, it should be noted that the first ultra-long acting β-2 stimulant, indacaterol, is currently the only LABA with a single daily administration and it has been demonstrated to be superior to other bronchodilators in reducing pulmonary hyperinflation [20] and dyspnea and increasing ability to exercise [21]. With regards to recent anticholinergics that enriched this segment, glycopyrronium (also a once-daily drug) demonstrated a notable improvement in endurance [11].

### Bronchodilators: how, where and when?

All guidelines for COPD declare bronchodilators as the baseline therapy for all stages of COPD. Instead, the choice of bronchodilator with which to start treatment is left up to the doctor in all guidelines (CTS, GOLD, AGE.NA.S, ACP/ACCP/ATS/ERS). Numerous systematic revisions have compared the efficacy of the treatment of inhalers for COPD with placebo and among themselves.

In an editorial written in 2011 by J.A. Wedzicha, while commenting the results of the POET study, it was argued that the initial therapeutic choice depends on the patient characteristics and thus LAMA in monodoses might be more indicated than LABA with the objective of reducing the number of exacerbations even if the comparison is unfair due to the fact that in that study tiotropium has a duration of 24 hours and salmeterol only 12. In fact, the comparison between uidtiotropium and ultra_LABAindacaterol shows that these drugs have comparable results for bronchodilation as well as symptoms, quality of life and relapses [22–24].

The combination of ICS/LABA compared with LAMAs has given contrasting results. For example, the exacerbations treated with steroids are more frequent in patients treated with LAMA, while in those using ICS/LABA treatments with antibiotics and pneumonia are more common, while hospitalizations were not significantly different in either of the two methods of treatment [25].

The analysis of the studies that compared LABA with ICS treatment confirms the current guidelines that foresee initial treatment with long-acting bronchodilation along with the addition of steroid inhalers for patients with frequent exacerbations [26].

On the other hand, regarding LABAs, there are those with 12 h (salmetorol and formetorol) or 24 h (ultra-LABA indacaterol) duration. Various studies have demonstrated that LABA reduce hospitalizations and dyspnea and increase lung function and quality of life. The safety profile of LABAswas comparable to placebo [27].

In general, long-acting bronchodilators have been proven to be able to produce a long-lasting improvement in lung function and quality of life and to reduce relapses in patients with COPD. They are also able to reduce hyperinflation and therefore to improve dyspnea and tolerance to physical activity [10, 28–32].

Moreover, it has been recently demonstrated that the co-formulation of LAMAs and LABAs improves the quality of life, increases FEV_1_, reduces dyspnea and relapses, compared to monotherapies (Shine study) [33].

The increasing number of drugs for COPD, both single and combined, increases on one side the number of therapeutical options, but on the other makes the choice more complicated. Further studies should be designed to give necessary evidence to the optimal sequence of monotherapy and the combination of bronchodilators to use in an algorithm of treatment for COPD [34–38].

### Role of anticholinergic bronchodilators: effectiveness and safety

Inhaled bronchodilators, with long-lasting action, are the most effective drugs in the regular treatment of COPD in the clinically stable phase. To control or improve symptoms and health, these are more effective and simpler to be used than short-acting bronchodilators, improving therefore the adherence to chronic therapy as well [39]**.**

The effectiveness of bronchodilators should be evaluated not only in terms of functional improvement (of the bronchial obstruction and pulmonary hyperinflation) but also in terms of relieve from symptoms (exercise tolerance and quality of life), and clinical improvement (reduction in number or severity of exacerbations), so that the absence of a spirometric response is not a reason for interrupting treatment, if there is subjective improvement in symptoms.

LAMAs (long-acting antimuscarinic agents) contrast excessive activity of the parasympathetic system, which has the fundamental function of regulating bronchial smooth muscular tone and prevents the inhalation of potentially irritating substances (such as atmospheric particles and cigarette smoke), thanks to a momentary increase in bronchial tone, with a consequent broncho-constriction. When, due to exogenous stimuli, the increase in bronchial tone becomes excessive and/or permanent, therapy with LAMA can be favorable.

Recent LAMAs act by impeding the bronchial obstruction for a prolonged period of time. The LAMAs have two characteristics, their binding to the receptor and kinetic selectivity, in other words, the capability to bind to- but also to leave- the receptor.

Clinically relevant muscarinic receptors are substantially of two types, M2 and M3. The M3 muscarinic receptors are variously located within the organism, but their concentration within the lung is particularly high. The M2 receptors are largely present in the cardiac muscle cells and, therefore, their prolonged stimulation could induce unwanted cardiological events. Selectivity in action is therefore capital.

Recently, Glycopyrronium bromide demonstrated to be an antagonist of long-action muscarinic receptors, which act by blocking the bronchoconstricting action mediated by the parasympathetic system and by the nervous fibers that release acetylcholine at the smooth muscle cell level in the airways. It showed a decisively greater selectivity (approximately 4 times) for human receptors M3 respect to M2 receptors [40].

Glycopyrronium has been evaluated in three phase III studies in which 2,700 patients were studied and it showed significant superiority compared to the placebo in improving respiratory function, measured with FEV_1_ (p <0.01), after 12 weeks of treatment. Glycopyrronium also showed a functional effectiveness (FEV_1_) similar to Tiotropium, during the 52 weeks of the GLOW2 study. Apart from demonstrating benefits for pulmonary function, Glycopyrronium showed a rapid onset of action, within 5 minutes from the first inhalation [41, 42].

The administration of the drug has also been shown to be effective in reducing the number of acute exacerbations of the illness.

Benefits have also been demonstrated with respect to the placebo, both for dyspnea and quality of life, measured with the TDI (Transition Dyspnea Index) scale and the SGRQ (St. George’s Respiratory Questionnaire), respectively. After the morning administration of Glycopyrronium, patients have benefitted from an improved resistance to physical activity, from the first doses on. In general, patients treated with Glycopyrronium presented an improvement in physical activity 21% higher than those with the placebo at the end of the study (day 21), with a significant increase of 10% from the first day (both p <0.001) [11].

In all the studies, patients, in the glycopyrronium-arm, demonstrated a drug-tolerance similar to that of the placebo, and the incidence of adverse events was also similar to the placebo [43, 44].

### Role of β-2 agonist bronchodilators: effectiveness and safety

β-2 agonist bronchodilators act mainly by relaxing the smooth muscle of the airways by binding with specific cellular receptors and through the activation of the adenylatecyclise [16]. For over 15 years the long-acting β-2 agonist bronchodilators (LABA) salmeterol and formoterol have been used in COPD therapy in stable phase. Both have a duration of approximately 12 hours [45] and they were demonstrated to be more effective that the short-acting β-2 agonist (SABA) in improving symptoms, respiratory function (FEV_1_), and tolerance to exercise in patients with COPD [46–48] even through the reduction of dynamic hyperinflation [49]. Various studies, including the mega-trial TORCH [50], have shown a reduction in the number of COPD exacerbations with the use of formoterol and salmeterol vs placebo [51, 52].

As already noted in studies of patients with bronchial asthma, formoterol produces a bronchodilation more rapidly than salmeterol [53]. However, there is still not sufficient data to prefer one of these LABA in maintenance therapy for COPD [54].

Indacaterol, the first “ultra-LABA” with an active duration of over 24 hours, has been available since 2010. Its characteristic allows mono- administration, which, along with a rapid onset of action, similar to formoterol and salbutamol, makes it potentially useful in increasing patient compliance [55]. The mechanism at the basis of indacaterol properties (rapid onset of action and persistence of effect) is still not fully clear; it is hypothesized that this depends on its peculiar interaction with the lipid membrane of the airways smooth muscle cells and on its greater capacity of diffusion in the lipophilic compartments after inhalation, with respect to previous generation of LABAs [56, 57].

Whatever the mechanism involved, indacaterol, when compared with various long-acting bronchodilators for the treatment of COPD, shows to be superior to formoterol and salmeterol with regards to effects on FEV_1_ and symptoms [58, 59] and comparable or superior to tiotropium when the duration of bronchodilation (trough FEV_1_), symptoms and quality of life have been considered [55, 60].

A recent meta-analysis of 20 randomized studies controlled with a placebo, conducted on a total of 8,774 patients with COPD, with a duration of at least 6 months (10 with formoterol, 9 with salmeterol and 4 with indacaterol) did not demonstrate differences in the major adverse events (in particular cardiovascular) between the group treated with LABA and the placebo. In 1% of patients there were events noted which are traditionally associated with the use of β-2 agonists (muscle tremors, palpitations), the systemic effects correlated to the stimulation of β-2 agonist receptors (hyperglycemia, hypokalemia) should be considered of a lesser clinical relevance [61]. However, a retrospective study of control cases in elderly COPD patients (over 67 years-old) that had developed severe cardiac arrhythmias (cases) vs those that had not (controls) demonstrated that the development of arrhythmias was more associated to the use of LABA (formoterol and salmeterol) with rate/ratio 1,47 [62]. So, as underlined in the guidelines, the use of LABA should always be carefully evaluated with attention in COPD patients with cardiovascular comorbidity or arrhythmias.

### Why and how to maximize bronchodilation

Because LABA and LAMA act via different mechanisms of action, they can exert additional bronchodilating effects, thus being able together to optimize and maximize pharmacologic bronchodilation in those COPD patients with severe airflow limitation, whose needs cannot be satisfied by monotherapy with a single, either LABA or LAMA bronchodilator. Therefore, current International and National guidelines (GOLD, AGE.NA.S, GARD) recommend the association of two long-acting bronchodilators when only one is not sufficient to provide satisfactory symptom relief [63]. In particular, indacaterol and glycopyrronium show to give very fast and long-lasting (about 24 hours) relaxation of airway smooth muscle [64–66]. These pharmacodynamic properties can provide, with a single daily administration, a stable and persistent bronchodilation avoiding fluctuations of bronchial calibre, in a way which has been colourfully defined as “pharmacological airway stenting”. On these grounds, the single-inhaler, indacaterol/glycopyrronium in fixed combination could be very effective in reducing dynamic lung hyperinflation as well as increasing compliance. We can assume that LABA and LAMA could exert their combined bronchodilating action also at the level of small airways, so counteracting one of the most important factors limiting exercise capacity in COPD such as air trapping, and decreasing dyspnea perception, and finally improving QoL. In addition, the sustained reduction in lung hyperinflation secondary to the decreased airway resistance, also given by LABA/LAMA associations, can also contribute to prevent COPD exacerbations [67]. In fact, improvements in lung hyperinflation are correlated with positive effects on frequency and severity of COPD exacerbations, probably due to a better re-setting of lung function dynamics elicited by long-acting bronchodilation.

The pharmacological basis of the reciprocal cooperation between LABA and LAMA is very strong. Indeed, LABA induce bronchodilation by relaxing airway smooth muscle regardless of the constricting stimuli, thus acting as functional antagonists of bronchoconstriction. On the other hand, LAMA inhibit the bronchoconstrictive effects of acetylcholine acting via a competitive antagonism of muscarinic receptors, thus complementing, integrating and potentiating the functional antagonism elicited by LABA [63]. As for selectivity, glycopyrronium exhibits a high kinetic selectivity for M_3_ versus M_2_ muscarinic receptors, while bronchodilator features depend on the powerful and prolonged blockade of M_3_ receptors, which in human airways are predominantly responsible for the bronchoconstrictive action of acetylcholine. Furthermore, the high degree of kinetic selectivity for M_3_ over M_2_ muscarinic receptors makes it possible for glycopyrronium to rapidly dissociate from M_2_ receptors, which within the airways inhibit the release of acetylcholine from postganglionic parasympathetic fibres. Therefore, the positive interactions between indacaterol and glycopyrronium extend from post-junctional, airway smooth muscle level, to pre-junctional, nerve terminal level, where the inhibitory effect on acetylcholine release mediated by stimulation of β2-adrenergic receptors converges with the analogous action exerted by activation of M_2_ muscarinic receptors, whose function is greatly spared by glycopyrronium. The therapeutic advantages of a fixed combination of LABA-LAMA, namely the one of indacaterol (110 μg)/glycopyrronium (50 μg), have been recently demonstrated by several clinical trials, including ENLIGHTEN, ILLUMINATE, SPARK and SHINE studies [33, 68–70], confirming what has been forecasted by guidelines as more effective of single-drug treatment.

### Safety of the co-formulation of LAMAs and LABAs

Combination of LAMAs and LABAs offer the potential of improved convenience and compliance over use of separate inhalers [71, 72]. The dose of each agent to be used in combination can be optimized. A major challenge in the development of combinations is provision of improved bronchodilation over monotherapy components while balancing the associated adverse events.

The safety profiles of both LAMAs and LABAs are well known. However, when combining the two drugs, it is important to understand both the similarities and differences in adverse events. Both LABAs and LAMAs can have effects on the cardiovascular system [73, 74]; these adverse events need to be monitored in development programs for combination products.

Results suggest that the cardiovascular safety profile of the fixed combination glycopyrronium/indacaterol (QVA149) is similar to placebo, with no clinically significant differences observed versus placebo [75].

In SHINE [33] and ENLIGHTEN [68] studies QVA149 was well tolerated over the 26 and 52 -week study, respectively, with an adverse events profile similar to that of placebo (Table 1). In addition, no actual or potential safety issues were observed with the combination compared with the single bronchodilators. The ILLUMINATE study compared the efficacy, safety, and tolerability of QVA149 versus salmeterol–fluticasone (SFC) over 26 weeks in patients with moderate-to-severe COPD, and the overall incidence of adverse events was similar for QVA149 and SFC treatment groups [70]. No untoward safety findings were apparent with the QVA149 approach compared with the single LAMA treatments (Glycopyrronium or Tiotropium) investigated in the SPARK study [69], all treatments were well tolerated and had acceptable profiles of cardio-cerebrovascular safety. Finally, the BEACON study [76] demonstrated that once-daily QVA149 provides an efficacy and safety profile similar to the concurrent administration of its monocomponents indacaterol and glycopyrronium.

**Table 1:** **Adverse events, serious adverse events, deaths and discontinuations over the 26-weeks treatment period**

	Placebo	QVA149110/50 μg	Indacaterol 150 μg	Glycopyrronium 50 μg	Tiotropium 18 μg
Subjects, n	232	474	476	473	480
Patients with any adverse events	134 (57.8)	261 (55.1)	291 (61.1)	290 (61.3)	275 (57.3)
COPD	91 (39.2)	137 (28.9)	153 (32.1)	150 (31.7)	138 (28.8)
Nasopharyngitis	23 (9.9)	31 (6.5)	35 (7.4)	46 (9.7)	40 (8.3)
Cough	8 (3.4)	26 (5.5)	38 (8.0)	18 (3.8)	21 (4.4)
Upper respiratory tract infection	13 (5.6)	20 (4.2)	32 (6.7)	20 (4.2)	24 (5.0)
Oropharyngeal pain	7 (3.0)	17 (3.6)	7 (1.5)	10 (2.1)	10 (2.1)
Viral upper respiratory tract infection	7 (3.0)	15 (3.2)	11 (2.3)	13 (2.7)	12 (2.5)
Upper respiratory tract infection bacterial	13 (5.6)	10 (2.1)	13 (2.7)	15 (3.2)	22 (4.6)
Lower respiratory tract infection	5 (2.2)	9 (1.19)	15 (3.2)	7 (1.5)	12 (2.5)
Back pain	5 (2.2)	8 (1.17)	11 (2.3)	17 (3.6)	8 (1.7)
Serious adverse events	13 (5.6)	22 (4.6)	26 (5.5)	29 (6.1)	19 (4.0)
Adjudicated CCV events					
Atrial fibrillation/flutter (new onset)	0	2 (0.4)	3 (0.6)	2 (0.4)	1 (0.2)
Serious CCV events	1 (0.4)	0	6 (1.3)	7 (1.5)	4 (0.8)
MACE	0	0	2 (0.4)	3 (0.6)	3 (0.6)

From [33].

### Combining LAMAs and LABAs: co-formulations

The current guidelines recommend the addition of a second bronchodilator to the initial therapy of moderate COPD, in order to maximize bronchodilation.

There have been clinical trials on the following combinations

- Formoterol + tiotropium
- Arformoterol or nebulized formoterol + tiotropium
- Salmeterol + tiotropium
- Indacaterol + tiotropium
- Indacaterol + glycopyrronium

Aaron and Coll, in 2007, published the results of the Optimal study, in which the effects of two bronchodilation treatments on FEV_1_were studied. The patients were divided in two groups: tiotropium + placebo in one group and tiotropium + salmeterol in the second group [77]. The FEV_1_ pre-broncodilator was shown to be superior in the tiotropium + salmeterol group, in all the evaluations, completed at 4, 20, 36, and 54 weeks. The group with double bronchodilation, furthermore, suffered a lower number of bronchitic relapses.

Similarly, there was a significant improvement in quality of life (SGRQ) with the double bronchodilation.

Mahler, in 2012, published data related to the INTRUST 1 and 2 studies, in which indacaterol 150 μguid + tiotropium 18 μguid was demonstrated to be significantly superior to tiotropium 18 μguid, evaluating both the standardized AUC of FEV_1_ from 5 minutes to 8 hours after the dose at the 12^th^ week, as well as the difference in FEV_1_ at the 12^th^ week [78].

Finally, Vincken et al. showed that in patients with moderate-to-severe COPD, once-daily coadministration of indacaterol and glycopirronium provides significant and sustained improvement in bronchodilation versus single broncodilator from day 1, with significant improvements in patient-centered outcomes [79].

After evaluating the results of the trials in which one bronchodilator was compared to the association of a beta adrenergic + an antimuscarinic, van der Molen in 2012 came to the following conclusions [80].

With respect to mono-therapy, the combined LABA + LAMA therapy:

- Improves breathing capacity and reduces hyperinflation
- Improves dyspnea
- Has a positive impact on the scores for evaluation of symptoms and on the use of rescue medications
- Is usually well tolerated

## Conclusions

The choice of an inhaled drug, perhaps more so than for other drugs, must take carefully into consideration three parameters: effectiveness, tolerability, and compliance.

After a close analysis of the scientific evidence, there is no doubt that bronchodilators have an essential role in the treatment of COPD.

Whether these are LABA, LAMA, or the combination of both, they are the first-line therapy to be used in fighting the progression of COPD as are, for example, inhaled corticosteroids in the treatment of an asthmatic patient.

In addition to the recent scientific evidence, the national and international guidelines and the recommendations for the use of the drugs, suggest using one or combined bronchodilators (LABA, LAMA or LABA + LAMA) as baseline therapy, drawing attention to the importance of maximizing bronchodilation.

To increase bronchodilation while simplifying adherence in COPD therapy a fixed combination could be the best option, which to-day is not available, unless the physician prescribes the two drugs separately.

In particular, indacaterol (ultra-LABA) and glycopyrronium (LAMA) are the most recent bronchodilators in monosomministration available and their pre-prepared combination (LABA/LAMA) has been approved by the European regulatory authorities in 2013. This has demonstrated statistically significant effectiveness on the key clinical outcomes, a good tolerability profile together with a good therapeutic compliance due to the single daily dose. While waiting for further confirmations, a fixed combination combining indacaterol (ultra-LABA) and glycopyrronium (LAMA) promises to be at the moment the only option to get optimal bronchodilation with uid administration in COPD.
